# New ways in delivering services for people with dual diagnosis

**DOI:** 10.1192/j.eurpsy.2024.329

**Published:** 2024-08-27

**Authors:** L. Lien, N. Halvorsen

**Affiliations:** ^1^ Norwegian psychiatric association; ^2^Norwegian psychiatric association, Norway, Oslo, Norway

## Abstract

**Introduction:**

People with severe mental health disorders and concurrent addiction problems are one of the most challenges patients to treat within mental health and addiction. They often find themselves fallen between different chairs within mental health and addiction services and between spescialist and primary care. There is a need for new ways of delivering services for this group.

**Objectives:**

The objective of this presentation is to present how Flexible assertive outreach teams (FACT) are delivered in a densly populated country and the results on changes in use of spescialist services and detension. We will also present the results of changes in quality of life before and after entering FACT and which factors that might be associated with life quality.

**Methods:**

The establising of FACT in Norway has been extecively evaluated both in the form of official reports to the health authorities and academic research papers. We will do a scoping review of the Norwegian research on the effect of FACT teams with a spesific attention to the results obtained in densly populated areas. The review will cover the years fra 2018 up til to day.

**Results:**

The results so far indicate that it is possible to deliver FACT services in densly populated areas and that there is an increase in qualty of life before and after entering a FACT team. The use of hospitalization days are reduced with about 50 % and the same applies for days in detention.

**Conclusions:**

FACT teams seems to be a viable way of delivering mental health care services to one of the most vulnerable groups in our society.

**Disclosure of Interest:**

None Declared

